# Perinatal Outcomes in Premature Placental Calcification and the Association of a Color Doppler Study: Report from a Tertiary Care Hospital in Eastern India

**DOI:** 10.3390/clinpract11040099

**Published:** 2021-11-09

**Authors:** Sudarshan Dash, Banya Das, Soumya Ranjan Panda, Monalisa Rajguru, Pramila Jena, Abheepsa Mishra, Sudhanshu Kumar Rath

**Affiliations:** 1 Department of Obstetrics and Gynaecology, Kalinga Institute of Medical Sciences, KIIT University, Bhubaneswar 751024, India; sudarshan.dash@kims.ac.in (S.D.); banyadas.parija23@gmail.com (B.D.); monalisha.rajguru@gmail.com (M.R.); pramila.jena@kims.ac.in (P.J.); sudhanshu.rath@kims.ac.in (S.K.R.); 2 IMO-JB, ESIC Hospital, Bhubaneswar 751022, India; 3 The Feinstein Institute for Medical Research, Manhasset, NY 11030, USA; mishra.abheepsa@gmail.com

**Keywords:** premature placental calcification, perinatal outcome, Doppler ultrasonography

## Abstract

**Introduction:** Placental calcification, identified before the 36th week of gestational age, is known as premature placental calcification (PPC). PPC could be a clue for the poor fetal outcome. However, its association with adverse perinatal outcomes is yet to be confirmed. **Objective:** The primary objective was to determine and compare the perinatal outcomes in pregnancies with and without documented premature placental calcification. **Methodology:** The present study was a prospective cohort study performed from October 2017 to September 2019. We consecutively enrolled 494 antenatal women who presented to our antenatal OPD after taking consent to participate in our study. Transabdominal sonographies were conducted between 28–36 weeks of gestation to document placental maturity. We compared maternal and fetal outcomes between those who were identified with grade III placental calcification (n = 140) and those without grade III placental calcification (n = 354). **Results:** The incidence of preeclampsia, at least one abnormal Doppler index, obstetrics cholestasis, placental abruption, and FGR (fetal growth restriction) pregnancies were significantly higher in the group premature placental calcification. We also found a significantly increased incidence of Low APGAR (Appearance, Pulse, Grimace, Activity, and Respiration) scores, NICU (Neonatal Intensive Care Unit) Admission, Abnormal CTG (cardiotocography), meconium-stained liquor, and low birth weight babies in those with grade III placental calcification. **Conclusion:** Clinicians should be aware of documenting placental grading while performing ultrasonography during 28 to 36 weeks. Ultrasonographically, the absence of PPC can define a subcategory of low-risk pregnant populations which probably need no referral to specialized centers and can be managed in these settings.

## 1. Introduction

Grade III placental calcification is a physiological aging process often found during term pregnancy [1,2,3]. It is known as premature placental calcification (PPC) when identified before the onset of the 36th week of gestation. PPC may be a clue for poor fetal outcome. However, the evidence for its association with the adverse feto-maternal outcomes seems ambiguous. Weinsberg and his group [4] first detected placental calcification with fetal maturity, followed by Granumm and his group [5], who first applied grading to the placenta. The prevalence of PPC is ranging from 3.8% to 23.7% in various studies [6,7]. Some studies reported the association of PPC with the incidence of FGR (fetal growth restriction), LBW (low birth weight) babies, and poor APGAR (Appearance, Pulse, Grimace, Activity, and Respiration) scores [6,8]. However, other investigators could not find such an association [1,2,3].

The varied results derived from these studies are confusing, which could be due to multiple causes. First, most of these studies were done years ago with ultrasound machines of lower resolution than the modern ones. Secondly, the study population was too small to provide a proper conclusion. Thirdly, cigarette smoking, diabetes, and hypertension were dismissed as confounding factors in a few studies. Hence, the current study aimed to address the grey areas on the feto-maternal outcomes of PPC-associated pregnancies.

### Objective

The primary objective was to determine and compare the perinatal outcomes in pregnancies with and without documented premature placental calcification.

## 2. Materials and Methods

### 2.1. Study Design

**Figure 1:**
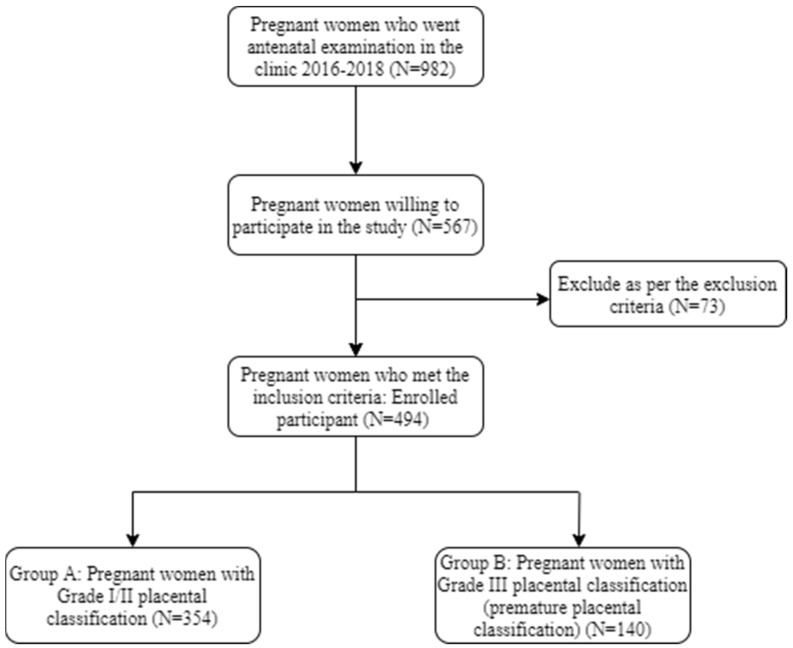
Flowchart showing participant selection criteria.

The prospective cohort study was performed between October 2017 to September 2019 after taking approval from the Institutional ethical committee of the hospital with EC registration number KIMS/KIIT/IEC/38/2017 dated 13 June 2017. After taking written informed consent, all antenatal women attending our OPD (Outpatient Department) were included in the study. The participants were enrolled (N = 494), and transabdominal sonographies were conducted between 28–36 weeks of gestation to document placental maturity. We compared maternal and fetal outcomes between patients identified with grade III placental calcification (n = 140) and those without grade III placental calcification (n = 354). Figure 1 shows the flowchart of the selection of participants for our study.

**Figure 2:**
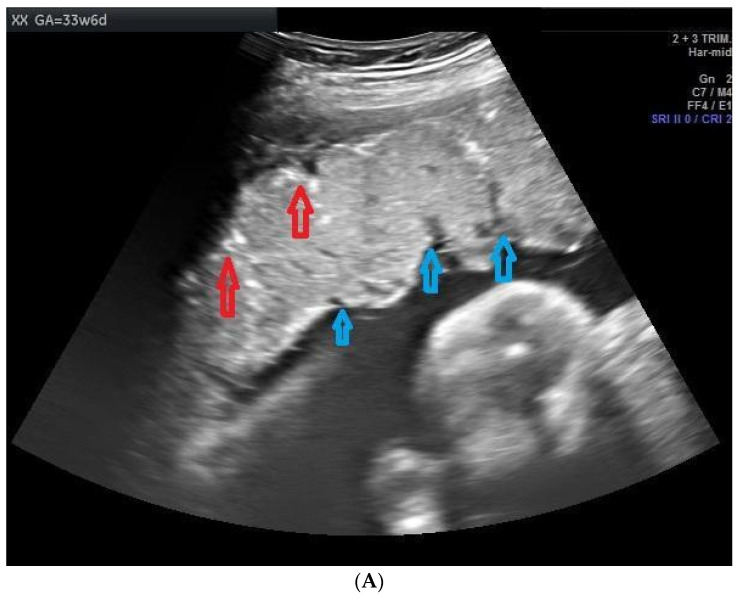

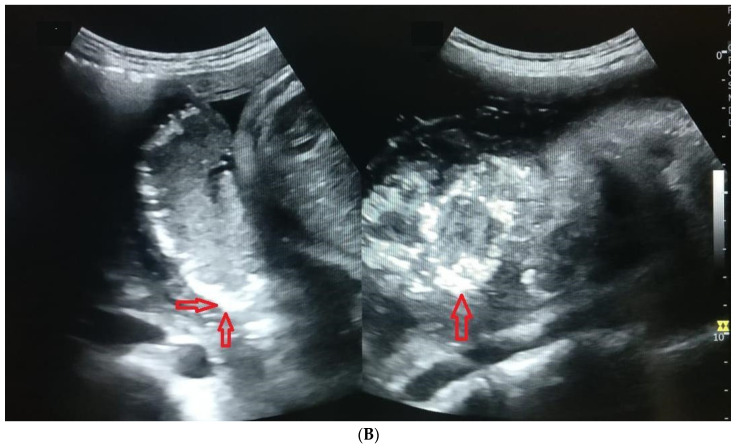
(**A**) Image showing placental calcification and lobulation (grade 3 placenta) in a case of 26-year-old primigravida at a gestational age of 33 weeks and 6 days. Red arrows show the placental calcification and blue arrows show lobulations. (**B**) Image showing grade 3 placental calcification in a 31-year-old primigravida at a gestational age of 35 weeks and 1 day. Red arrows show the placental calcification.

Ultrasound was performed to document the cases of PPC from 28 weeks of gestation at every four-week interval. Echogenic indentation from the chorionic plate to the basal layer dividing the placenta into random lobules, similar to cotyledons, was considered Grade III placental calcification (Figure 2A,B). A doppler flow study was conducted starting from 32 weeks of gestation to 36 weeks at an interval of 4 weeks. The ultrasound equipment used for this study was a Siemens Acuson × 300 PE USG machine with a curvilinear transducer and a frequency of 1.9 to 6.4 MHz, analyzed by a single consultant in order to avoid interobserver bias. The images were verified by another senior consultant to ensure the accuracy of diagnosis. Both the consultants were obstetricians trained in ultrasonography. In the umbilical artery Doppler study, absent or reversed end-diastolic velocity (AREDV) was considered to reflect poor uteroplacental blood flow. The other Doppler indices measured were the umbilical artery pulsatility index (Umb PI) ≥ 95th, middle cerebral artery pulsatility index (MCA PI ≤ 5th), cerebro placental ratio (CPR ≤ 5th), Mean uterine PI ≥ 95th, and unilateral or bilateral notch in the uterine artery.

The study participants were divided into two groups based on the presence or absence of premature calcification. Each pregnancy was followed until six weeks postpartum, and in each case, antepartum, intrapartum, and postpartum complications were documented. A comparison was made between two groups for feto-maternal outcome and indices of Doppler velocimetry of the uteroplacental blood flow.

Preeclampsia was defined as raised blood pressure (>140/90 mm of Hg) found between the 20th week of gestation and 6th week of post-partum. Gestational diabetes was defined as carbohydrate intolerance found after the 20th week of gestation as per an oral glucose tolerance test done after the 24th week of gestation. Placental abruption was suspected clinically if patients developed antepartum hemorrhage after the 28th week of gestation and ultrasonography showing a placenta in its normal location but showing signs of a retroplacental hemorrhage/clot.

Inclusion criteria: All pregnant women attending our antenatal OPD after 28 weeks of gestation were included in this study. 

Exclusion criteria: Women with multifetal gestation, congenital anomalies of the fetus, history of smoking and alcohol consumption, previous history of diabetes mellitus, hypertension, and those who did not consent to participate were excluded from the study.

### 2.2. Statistical Analysis

The data were tabulated and expressed as mean ± SD or frequency and percentage for continuous and categorical variables, respectively. Chi-square or Fisher Exact test was used to determine the association between two categorical variables. The student’s *t*-test was performed to test the significance of the difference between the two groups. All statistical calculations were performed using the SPSS software version 21, and a *p*-value ≤ of 0.05 was considered statistically significant.

## 3. Results

A total of 494 pregnant women were included in our analysis. Out of these, 354 (71.7%) did not show any evidence of PPC (group A) and 140 (28.3%) did show evidence of PPC (group B). The two groups were similar in context with the baseline characteristics (Table 1). The maternal and perinatal outcomes of the two groups are compared in Table 2 and Table 3, respectively. The incidence of preeclampsia (16.7% versus 28.6%; *p*-value-0.002), at least one abnormal Doppler index (20.9% versus 35.7%; *p*-value-0.001), obstetrics cholestasis (6.8% versus 14.3%; *p*-value-0.008), placental abruption (8.1% versus 17.1%; *p*-value-0.004), and FGR (11% versus 21.4%; *p*-value-0.002) were significantly higher in the group having PPC. These parameters bear a relative risk (95% CI) of 1.71 (1.2074 to 2.4339), 1.70 (1.2647 to 2.3080), 2.1 (1.2033 to 3.6898), 2.09 (1.2639 to 3.4647), and 1.94 (1.2604 to 3.0017), respectively for preeclampsia, at least one abnormal Doppler index, obstetrics cholestasis, placental abruption, and FGR (Table 2 and Figure S1). At the same time, we did not find any statistical difference between the incidence of GDM (gestational Diabetes Mellitus), PPROM (preterm premature rupture of membranes), maternal anemia, hemoglobinopathies, postpartum hemorrhage, and mode of delivery between the two groups.

As far as neonatal outcome is concerned, we found a significantly increased incidence of Low APGAR (6.8% versus 14.3%; *p*-value-0.004), NICU (Neonatal Intensive Care Unit) admission (4.2% versus 7.1%; *p*-value-0.026), abnormal cardiotocography (11% versus 18.5%; *p*-value-0.024), meconium-stained liquor (8.7% versus 17.8%; *p*-value-0.004), and low birth weight (LBW) babies (24.01% versus 32.8%; *p*-value-0.04) in those with PPC. These bear a relative risk (95% CI) of 2.10 (1.2639 to 3.4647), 2.10 (1.0927 to 4.0634), 1.68 (1.0684 to 2.6597), 2.03 (1.2503 to 3.3258), and 1.36 (1.0130 to 1.8484), respectively. However, the difference was not significant between the two groups as far as preterm birth or neonatal death is concerned (Table 3 and Figure S2).

Comparison of Doppler parameters between the two groups is shown in Table 4. As one can notice, parameters such as UMB PI ≥ 95th, MCA PI ≤ 5th, CPR ≤ 5th, and AREDF had significantly worse values in the group of pregnant women with PPC. The indications of LSCS are shown in Table 5.

The performance of PPC is shown in Table 6. This shows that for most of the adverse perinatal outcomes, the negative predictive value and specificity of PPC is very good. The negative predictive value of PPC is highest for NICU admission (94.9%), followed by Low APGAR (91.8%), placental abruption (91.8%), Meconium-stained liquor (91.2%), FGR pregnancies (89%), and abnormal Doppler parameters (79.1%). Similarly, the specificity was found to be highest for abnormal Doppler parameters (75.7%) followed by preeclampsia (74.7%), FGR pregnancies and LBW babies (74.1%), Low APGAR (73.7%), placental abruption (73.7%), Meconium-stained liquor (73.7%), abnormal CTG (73.4%), and NICU admission (72.9%).

## 4. Discussion

In our study, premature placental calcification was associated with adverse maternal and neonatal outcomes, such as preeclampsia, at least one abnormal Doppler index, obstetrics cholestasis, placental abruption, FGR, maternal ICU admission, low birth weight babies, and low neonatal APGAR scores.

PPC can cause gradual narrowing of placental vessels due to the deposition of calcium and fibrin, leading to decreased uteroplacental blood flow. This way, it can cause an adverse feto-maternal outcome. There are two possible explanations for our findings [9]. In fact, as evidence for this hypothesis, widespread basement membrane mineralization [10], focal calcification, and acute atherosclerosis in the placental vessels [11] were found on pathologic examination of the placentas in fetal Bartter syndrome. A similar case was reported where massive calcification and thrombi, including the chorionic and umbilical vessels, were the cause of severe fetal growth restriction [12].

Another hypothesis for PPC leading to poor uteroplacental blood flow and adverse feto-maternal outcomes could be via an unknown root cause that had remained uninvestigated. However, there is no direct research supporting this theoretical view. We theorize that myometrial contraction causing tissue separation in the interface contributed by hormones could lead to early detachment. The process could be mediated via some unknown pathway in the placenta involved with preterm calcification. These events are pretty similar to the separation of deciduas spongiosa and the formation of a retroplacental hematoma at term [13].

According to our study, in high-risk pregnant women, premature placental calcification is a pathological event that may have a different mechanism from that of placental calcification found at term. It was observed that the disorders of calcium pumps located at the placental basement membrane [14] could play a significant role in excessive calcium deposition in the placenta. This can lead to marked calcification of the placental basement membrane. There is also evidence that nanobacteria play a central role in early pathologic calcification, albeit in limited cases [15,16]. Although a detailed analysis of the calcification cascade is out of context for this discussion, future research should explore such relationships. Similar to our study, some other authors have also found preterm placental calcification to be associated with a greater incidence of fetal growth restriction [6,7,17,18,19], low birth weight [6,7,18,19,20,21], low APGAR scores [18], and pregnancy-induced hypertension [6,17,19,22]. On the other hand, some other authors did not find any association of preterm placental calcification with fetal growth restriction [23,24], low birth weight [25], and low APGAR scores [25]. As suggested by the Euronatal audit study, there might be a reduction in the incidence of stillbirth rates with improvements in the detection and management of growth-restricted fetuses [26]. In their study, Mirza and his group found a significant association of PPC with low-birth-weight babies and perinatal death [27]. In line with our study, other recent studies also found PPC is associated more commonly with abnormal uterine and Umbilical Artery Doppler indices [8,28].

According to our study, the negative predictive value and specificity of PPC for predicting poor perinatal outcome is very high. This indicates that absence of PPC in any pregnant women can be extremely useful in low resource settings to identify a subcategory of low-risk pregnant women who may not need any future referral to specialized centers. Thus, these pregnancies can be safely managed in these particular places. On the other hand, the presence of PPC may warrant clinicians to perform Doppler ultrasound evaluation of placenta and fetus in such pregnancies.

### Strengths and Limitations

The strengths of this study lies in the fact that it is a well-designed prospective cohort study. However, certain limitations should be considered while analyzing the results of the study. First, our study was performed in a tertiary care hospital. Applying the conclusions to low-resource settings is not necessarily valid because of questionable external validity. Second, the placental histology was not performed to confirm the placental calcification found on ultrasonography. However, to be specific, the parameter we intended to evaluate initially was ultrasonographically diagnosed PPC or premature grade 3 placental maturity. Thus, there was no need to do a placental histology to corroborate the ultrasound finding. Third, the performance of PPC could have been better characterized in a larger sample size.

## 5. Conclusions

Premature placental calcification should be considered as one of the reasons for underlying placental dysfunction and not as a mere physiological aging process. It is associated with increased maternal complications as well as increased adverse neonatal outcomes. Hence, these women should be carefully investigated with antepartum surveillance for fetal wellbeing and monitored for maternal complications. We suggest that clinicians be aware of documenting placental grading while performing ultrasonography during 28 to 36 weeks. This is especially true for low resource settings, where the absence of PPC can define a subcategory of low-risk pregnant populations, which probably need no referral to a specialized center and can be managed in these settings. However, future well-controlled prospective studies with large sample sizes are required to better characterize the performance of PPC as a predictor of poor feto-maternal outcome.

## Figures and Tables

**Table 1:** Characteristics of women in the two study groups.

Characteristics	Group A (Those without Premature Placental Calcification) n = 354	Group B (Those with Premature Placental Calcification) n = 140	*p*-Value
Maternal age (y)	26.79 ± 2.05	26.36 ± 2.34	>0.05
Body mass index (kg/m^2^)	23.9 ± 2.05	24.3 ± 1.95	>0.05
Socio-Economic status			
Lower Lower middle Upper middle	82 (23.1%) 192 (54.2%) 80 (22.7%)	36 (25.7%) 71 (50.7%) 24 (23.6%)	>0.05
Parity			
Primigravida Multigravida	148 (41.8%) 206 (58.2%)	48 (34.2%) 92 (65.8%)	>0.05

**Table 2:** The maternal outcome for the selected cohort of pregnant women.

	Total n = 494	Group A (Those without Premature Placental Calcification) n = 354	Group B (Those with Premature Placental Calcification) n = 140	*p*-Value	Relative Risk (95% CI)
Pre-eclampsia	99 (20.0)	59 (16.7)	40 (28.6)	0.002	1.71 (1.20 to 2.43)
GDM	58 (11.7)	40 (11.3)	18 (12.8)	0.626	1.13 (0.67 to 1.91)
At least one abnormal Doppler index (uterine or umbilical or fetal middle cerebral arterial index) *	124 (25.1)	74 (20.9)	50 (35.7)	0.001	1.70 (1.26 to 2.30)
PPROM	147 (29.7)	97 (27.4)	50 (35.7)	0.063	1.30 (0.98 to 1.72)
ANEMIA	25 (5.1)	15 (4.2)	10 (7.1)	0.1871	1.68 (0.77 to 3.66)
Obstetrics cholestasis	44 (8.9)	24 (6.8)	20 (14.3)	0.009	2.1 (1.20 to 3.68)
FGR	69 (14.0)	39 (11.0)	30 (21.4)	0.002	1.94 (1.26 to 3.00)
Hemoglobinopathy	15 (03.0)	10 (2.8)	5 (3.6)	0.663	1.26 (0.44 to 3.63)
Placental abruption	53 (9.3)	29 (8.1)	24 (17.1)	0.004	2.09 (1.26 to 3.46)
PPH	45 (9.1)	31 (8.75)	14 (10)	0.664	1.14 (0.62 to 2.08)
Maternal transfer to ICU	78 (15.7)	49 (13.8)	29 (20.7)	0.057	1.49 (0.98 to 2.26)

* Doppler indices measured were UMB PI ≥ 95th, MCA PI ≤ 5th, CPR ≤ 5th, Mean uterine PI ≥ 95th and unilateral or bilateral notch in uterine artery. GDM: Gestational Diabetes Mellitus; PPROM: preterm premature rupture of membranes; PPH: post-partum hemorrhage; FGR: Fetal growth restricted pregnancies; Umb PI, Umbilical pulsatility index; MCA, Middle cerebral artery; CPR, Cerebroplacental ratio.

**Table 3:** Perinatal outcome for the selected cohort of pregnant women.

	Total (n = 494)	Group A (Those without Prematureplacental Calcification) n = 354	Group B (Those with Premature Placental Calcification) n = 140	*p*-Value	Relative Risk (95% CI)
Preterm birth	70 (14.2)	45 (12.7)	25 (17.9)	0.139	1.40 (0.89 to 2.19)
Low APGAR	53 (08.9)	29 (6.8)	24 (14.3)	0.004	2.10 (1.26 to 3.46)
NICU Admission	33 (05.1)	18 (4.2)	15 (7.1)	0.026	2.10 (1.09 to 4.06)
Abnormal CTG	65 (13.15)	39 (11.0)	26 (18.5)	0.024	1.68 (1.06 to 2.65)
Meconium-stained liquor	56 (11.3)	31 (8.7)	25 (17.8)	0.004	2.03 (1.25 to 3.32)
LBW	124 (25.1)	85 (24.01)	46 (32.8)	0.040	1.36 (1.01 to 1.84)
Birth weigt (g)	2714.25 + 496.62	2575.04 + 606.75	0.008		
Gestation at delivery	38 wk 3 d + 1 wk 5 d	38 wk 0 d + 1 wk 6 d	0.056		
Delivery Mode (LSCS)	172 (34.8)	118 (33.3)	54 (38.5)	0.263	1.11 (0.89 to 1.49)
Neonatal death	15 (03.0)	10 (2.8)	5 (3.6)	0.663	1.26 (0.44 to 3.63)

NICU: neonatal intensive care unit; LBW: low birth weight babies; CTG: Cardiotocography; LSCS: lower segment caesarean section.

**Table 4:** Comparison of Doppler abnormalities between the two groups.

Doppler Characteristics	Group A (Those without Premature Placental Calcification) n = 354 (%)	Group B (Those with Premature Placental Calcification n = 140 (%)	*p*-Value
UMB PI ≥ 95th,	32 (9.03)	27 (19.2)	0.003
MCA PI ≤ 5th,	29 (8.19)	25 (17.8)	0.003
CPR ≤ 5th,	27 (7.62)	20 (14.2)	0.027
Mean uterine PI ≥ 95th	26 (7.34)	18 (12.8)	0.078
Unilateral notch in uterine artery.	35 (9.88)	20 (14.2)	0.203
bilateral notch in uterine artery.	22 (6.21)	9 (6.4)	1.000
AREDF	15 (2.82)	13 (5.71)	0.049
At least one abnormal Doppler index (uterine or umbilical or fetal middle cerebral arterial index) *	74 (20.9)	50 (35.7)	0.001

* Doppler indices measured were UMB PI ≥ 95th, MCA PI ≤ 5th, CPR ≤ 5th, Mean uterine PI ≥ 95th, unilateral or bilateral notch in uterine artery and AREDF. Umb PI, Umbilical pulsatility index; MCA, Middle cerebral artery; CPR, Cerebroplacental ratio; AREDF: absent/ reversed end diastolic flow.

**Table 5:** Indications of LSCS.

Indications	Group A (Those without Premature Placental Calcification) n = 354 (%)	Group B (Those with Premature Placental Calcification n = 140 (%)
CPD	25 (7.06)	12 (8.57)
Fetal Distress	29 (8.19)	16 (11.4)
CDMR	24 (6.77)	10 (7.14)
Antepartum hemorrhage	10 (2.82)	4 (2.85)
Obstructed Labour	6 (1.69)	2 (1.42)
Severe preeclampsia	24 (6.77)	10 (7.14)

**Table 6:** Performance of premature placental calcification.

	Sensitivity (%)	Specificity (%)	Positive Predictive Value (%)	Negative Predictive Value (%)
preeclampsia	40.4	74.7	28.6	83.3
FGR	43.5	74.1	21.4	89
PLACENTAL ABRUPTION	45.3	73.7	17.1	91.5
Low APGAR	45.3	73.7	17.1	91.5
NICU admission	45.5	72.9	10.7	94.9
Abnormal CTG	40	73.4	18.6	89
Meconium-stained liquor	44.6	73.7	17.9	91.7
LBW	35.1	74.1	32.9	76
Abnormal Doppler parameters	40.3	75.7	35.7	79.1

## Supplementary Materials

The following are available online at https://www.mdpi.com/article/10.3390/clinpract11040099/s1, Figure S1: The maternal outcome for the selected cohort of pregnant women, Figure S2: Perinatal outcome for the selected cohort of pregnant women.
