# Supplementary material for: Perinatal Outcomes in Premature Placental Calcification and the Association of a Color Doppler Study: Report from a Tertiary Care Hospital in Eastern India
Source: Clin Pract. 2021 Nov 9;11(4):841–9. doi: 10.3390/clinpract11040099 (PMC8628690; doi:10.3390/clinpract11040099)

Supplementary Information

Figure S1: The maternal outcome for the selected cohort of pregnant women

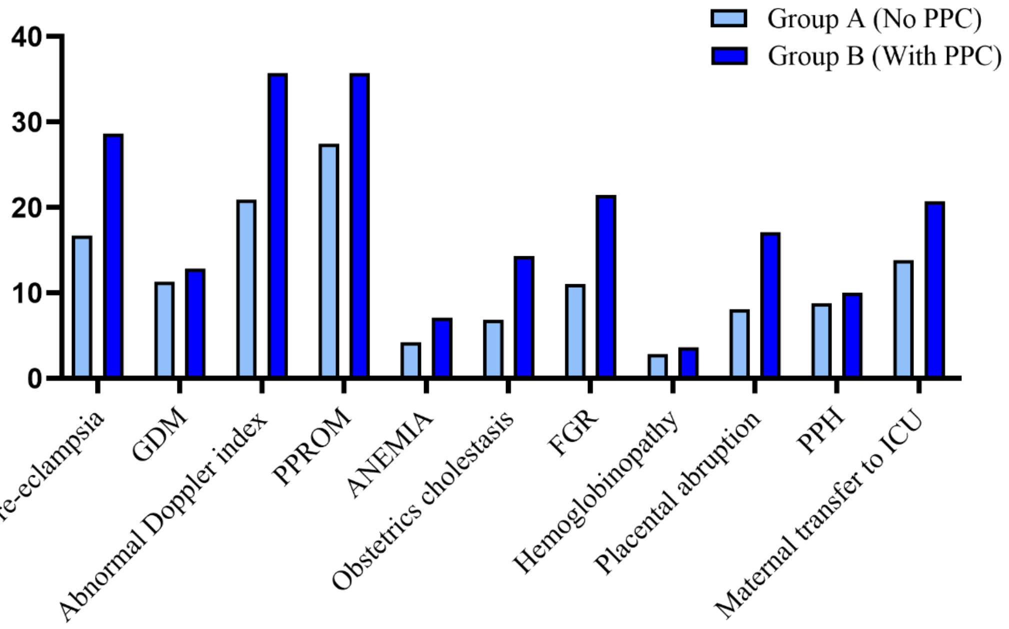

Figure S2: Perinatal outcome for the selected cohort of pregnant women.

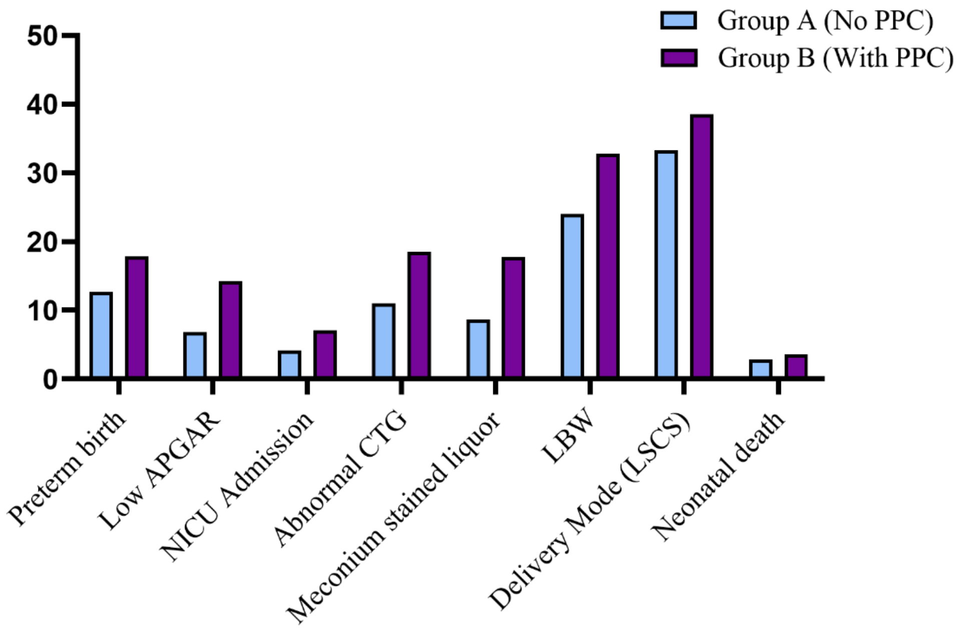

Supplement: Supplementary file 1 [file clinpract-11-00099-s001.zip › clinpract-1387009-supplementary.pdf]
